# TiO_2_ as Photosensitizer and Photoinitiator for Synthesis of Photoactive TiO_2_-PEGDA Hydrogel Without Organic Photoinitiator

**DOI:** 10.3389/fchem.2018.00340

**Published:** 2018-08-07

**Authors:** Sarah Glass, Betsy Trinklein, Bernd Abel, Agnes Schulze

**Affiliations:** Leibniz Institute of Surface Engineering (IOM), Leipzig, Germany

**Keywords:** hydrogels, titania, biomaterial, photopolymerization, photodynamic therapy

## Abstract

The replacement of potentially toxic photoinitiators is of increasing interest regarding the synthesis of biomaterials by photopolymerization. Therefore, we present a new method for the preparation of UV polymerized hydrogels with TiO_2_ as a photoinitiator. Titania is known to be an excellent photoactive compound which is non-toxic, inert, and cheap. The so-formed hydrogels possess excellent mechanical properties, a high swelling ratio, and high thermal stability. Furthermore, no TiO_2_ is released from the hydrogels. Thus, the material is highly suitable for medical applications. Additionally, the present TiO_2_ in the hydrogels remains photoactive as demonstrated by degradation of methylene blue. This enables the application of TiO_2_-hydrogels in photodynamic therapy.

## Introduction

Hydrogels are three-dimensional polymeric networks made out of highly hydrophilic monomers (Wichterle, 1960; Hoare and Kohane, 2008). They can be produced from natural materials like chitosan, dextran, and gelatin (Cabral et al., 2014; Wisotzki et al., 2014; Chan et al., 2015), or from artificial monomers [e.g., poly(ethylene glycol), polyamides, and several more] (Fujishige and Ando, 1989; Lin and Anseth, 2009). Mixtures of both are also possible (Jiang et al., 1999). Of course, the choice of material influences its properties (Ahmed, 2015) and therefore its application. Typical applications for hydrogels are artificial tissues (Lee and Mooney, 2001), wound dressings (Frykberg and Banks, 2015), contact lenses, drug delivery systems (Hoare and Kohane, 2008) and several other biomedical applications (Peppas et al., 2000; Ahmed, 2015; Caló and Khutoryanskiy, 2015; Chai et al., 2017).

Hydrogels are very often synthesized by photopolymerization (Peppas and Khare, 1993; Fairbanks et al., 2009). Herein a photoinitiator is used which produces reactive species, such as radicals, when irradiated with light. These radicals can initiate polymerization reactions for hydrogel synthesis. In general, a non-cytotoxic and biocompatible material is required for biomedical applications. Nevertheless, some photoinitiators are known to be cytotoxic (Williams et al., 2005; Mironi-Harpaz et al., 2012; Xu et al., 2015). The toxicity of photoinitiators is usually dependent on the concentration and exposed tissue (Sabnis et al., 2009). Consequently, crosslinking efficiency and cytotoxicity have to be considered (Mironi-Harpaz et al., 2012). Therefore, an alternative biocompatible photoinitiator is preferable. A promising photoinitiator candidate could be TiO_2_ (titania), which has been used as an inorganic photoinitiator for materials synthesis in previous studies (Wan et al., 2006; Liao et al., 2014; Lobry et al., 2016).

Titania is a well-studied photoactive compound with excellent mechanical, optical, and physical properties (Kazuhito et al., 2005; Chen and Mao, 2007; Gupta and Tripathi, 2011). Furthermore, TiO_2_ possesses high chemical stability, superhydrophilicity (Gan et al., 2007), biocompatibility (López-Huerta et al., 2014), reusability, and can be produced at low cost (Kumar and Bansal, 2013). Therefore, it has been investigated regarding several applications such as water purification (Kumar and Bansal, 2013), antifogging (Gan et al., 2007), disinfectant (Kumar and Bansal, 2013), and anticancer therapy (Lee et al., 2010) in the last centuries (Kazuhito et al., 2005).

TiO_2_ is a semiconductor and has a band gap of 3.2 eV (λ < 390 nm). It exists in an amorphous phase that is non-photoactive (Henderson, 2011). Furthermore, there are three natural crystalline modifications: anatase, rutile, and brookite (Gupta and Tripathi, 2011). Rutile and anatase have been especially investigated since the 1950s (Kazuhito et al., 2005). Anatase was found to be more photoactive than rutile and brookite (Auguliaro et al., 1990; Zhang et al., 2014). Therefore, anatase is favored in photocatalytic applications. The composition of titania is dependent on various conditions during the preparation such as temperature (Gupta and Tripathi, 2011), pressure, and others (Fischer et al., 2017). A well-known, commercially available TiO_2_ composite to be mentioned here is Degussa P25. Here, the anatase to rutile ratio is 70:30.

Given the fact that TiO_2_ has such outstanding properties, it is widely used in various (hybrid) materials. Typical examples are concretes (Qin et al., 2015), ceramics (Bianchi et al., 2017), and also polymeric materials like membranes (Fischer et al., 2017) and hydrogels (Zhang et al., 2013). Such polymeric materials may also be used for photocatalytic or photomedical applications like photodynamic therapy (PDT). In PDT, so called photosensitizers (either organic dyes or photoactive pigments) are employed. A photosensitizer can produce singlet oxygen or other reactive oxygen species like hydroxyl radicals when irradiated with light (Raab, 1900). For several years this mechanism has been used very effectively in photomedicine (Rehman et al., 2016) because these reactive oxygen species are highly toxic against cancer cells (Dolmans et al., 2003). Moreover, photosensitizers have been used for the treatment of bacteria, viruses and fungi for some years (Juzeniene et al., 2007). Consequently, they are used in non-oncologic applications like infected or badly healing wounds.

**Figure 1:**
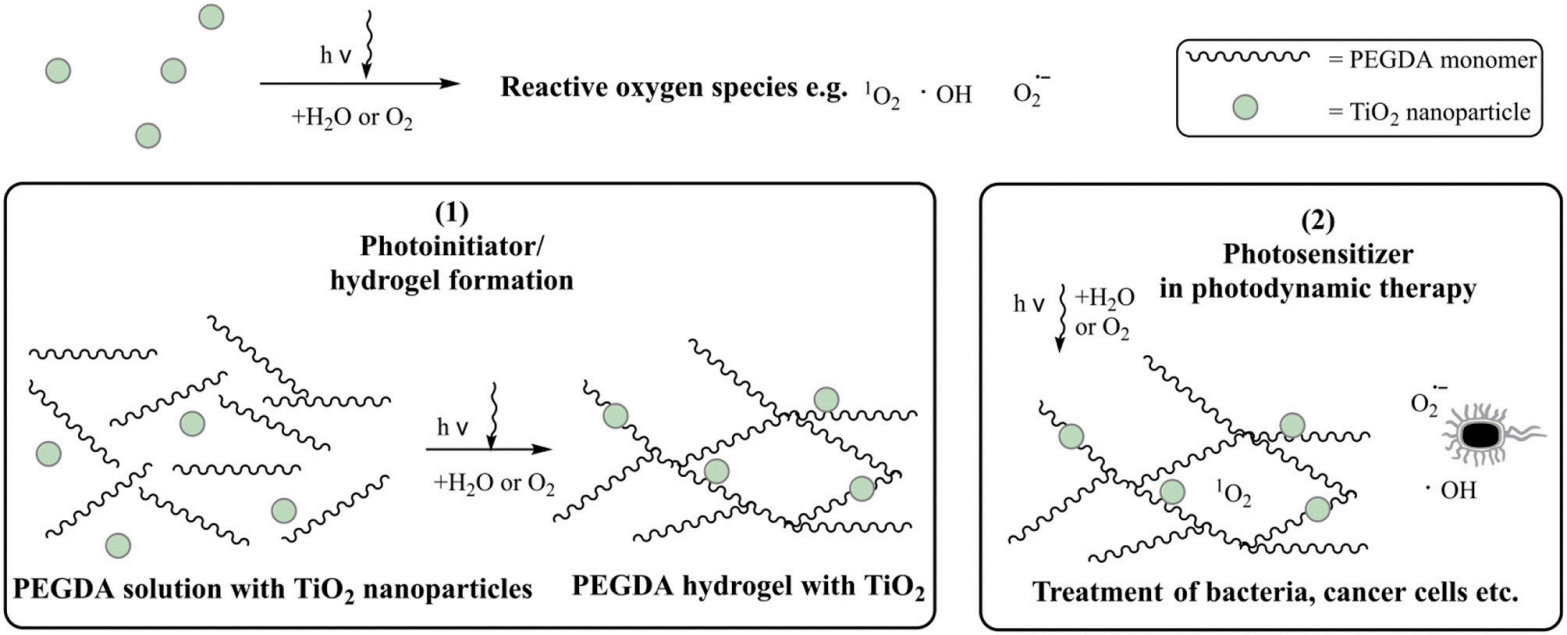
Schematic graphic of the usage of TiO_2_ nanoparticles as (1) photoinitiator and (2) photosensitizer.

As a result, TiO_2_ is an ideal candidate as a photoinitiator and photoactive compound in polymeric hybrid materials. In the present study, TiO_2_ was used with two functions at the same time as shown in Figure 1:

1. photoinitiator: initiation of the photopolymerization to prepare the biocompatible hydrogel
2. photosensitizer: formation of singlet oxygen/reactive oxygen species as a possible application in photodynamic therapy.

A hydrogel prepared with TiO_2_ remains photoactive, and because no organic photoinitiator is used it is non-cytotoxic. Because of the mechanical properties of the hydrogel it can also be applied as a wound dressing or bandage. Furthermore, this new method enables, in general, the synthesis of materials for biomedical applications.

## Material and methods

### Materials

Poly(ethylene glycol)diacrylate with an average mass of 700 g mol^−1^ (PEGDA), titanium isopropoxide (97% in isopropanol, TTIP), 37 wt% hydrochloric acid, nutrient solution, fetal bovine serum and phosphate buffer saline (PBS) were purchased from Sigma-Aldrich (St. Louis, USA). A stock solution of 50 wt% PEGDA in water was used to prepare the formulations. 1-[4-(2-hydroxyethoxy)-phenyl]-2-hydroxy-2-methyl-1 propane 1-one (α-HAP) was bought from IGM Resins B.V. (Waalwijk, Netherlands). Resazurin solution (Alamar blue) was purchased from Thermo Fisher Scientific (Waltham, USA).

A Merck ultrapure water system was used to purify water. All chemicals were used without further purification.

### Preparation of crystalline TiO_2_-nanoparticles

The TiO_2_ nanoparticle synthesis follows the method recently employed for polymer membranes (Fischer et al., 2017). In that process, 8.1 ml TTIP was slowly added to 80 ml of 0.1 M hydrochloric acid. The mixture was transferred to a temperature- and pressure-safe Teflon beaker and was shaken at 300 rpm for 4 h. Afterwards, it was heated to 60°C for 20 h. Once cooled down, the clear liquid on top of the solution was removed and 22.5 ml of water were added. An opaque and white suspension was received. The amount of TiO_2_ in the solution was determined gravimetrically to be 5 wt%. The solution was used within 2 days after preparation to avoid agglomeration.

### XRD

The TiO_2_ particles were dried at room temperature and crushed to a fine powder. X-ray diffractograms of these nanoparticles were recorded with a Rigaku Ultima IV X-ray diffraction spectrometer with Cu Kα radiation with the following parameters: 40 kV, 40 mA, scanning speed of 1.0° min^−1^, and in increments of 0.02° in a 10–75° range.

The weight fractions (W) of anatase (A), brookite (B), and rutile (R) were calculated from the peak integrals by using the following equation, where A corresponds to the peak areas and k corresponds to the XRD response factors of the respective crystal phases:

(1) $$W_{\left(A,R,B\right)}=\;\frac{k_{\left(A,R,B\right)}\cdot A_{\left(A,R,B\right)}}{k_{A}\cdot A_{A}+k_{R}\cdot A_{R}+k_{B}\cdot A_{B}}$$

The response factors were set to (k_A_ = 0.886, k_R_ = 1 and k_B_ = 2.721) (Liu et al., 2016).

### Hydrogel synthesis

All hydrogels were prepared with 30 wt% PEGDA, PBS, and a photoinitiator. The formulations were sonicated for 15 min and afterwards irradiated with a medium pressure mercury lamp (UV Technik Meyer GmbH, Germany) on a conveyor. The light dose corresponded to 7,000 mJ cm^−2^ and 2,500 mJ cm^−2^ for the TiO_2_ gels and the α-HAP gels, respectively. Afterwards, the gels were washed with PBS for 24 h and then shaken in water for 2 h at 200 rpm. Finally, the gels were dried at 40°C for 24 h in a N_2_ atmosphere. The hydrogels were synthesized either with 1 wt% crystalline TiO_2_ or with 0.1 wt% of a commercial initiator (α-HAP) as a reference (Pelras et al., 2017). α-HAP was used because it is also known to be cytocompatible (Williams et al., 2005).

### UV/VIS spectroscopy

An UV-2101PC UV-Vis spectrometer from Shimadzu was used to record transmittance curves of the dried hydrogels. The studied range was 200–800 nm with 0.5 nm resolution. Samples had an average thickness of 1 mm.

### Rheology

Rheological measurements of the dried hydrogels were performed on a MCR300 rheometer from Anton Paar (Graz, Austria). The rheometer was equipped with a planar 25 mm diameter head. The frequency was varied from 0.16 to 16 Hz. The probe head was pressed on the sample with a pressure of 10 N.

### Swelling ratio

The swelling ratio q was calculated as a ratio of the mass of the wet hydrogel directly after preparation and washing (m_wet_), to the mass of the dry hydrogel (m_dry_) as shown in equation (2). Swelling ratios are given in percent.

(2) $$q=\;\frac{m_{wet}}{m_{dry}}$$

### Scanning electron microscopy

The freshly dried hydrogels were sputtered with a 30 nm chromium layer using a Leybold Z400 sputter system. Afterwards, scanning electron microscopy (SEM) was performed using an Ultra-55 microscope equipped with a Gemini Detector both from Zeiss (Jena, Germany) at a pressure of 2 × 10^−5^ mbar and 5 kV voltages. The top, bottom, and cross-section of all gels were analyzed.

### Release study

The dried hydrogels were immersed in 10 mL PBS buffer at 37°C. The TiO_2_ particles were allowed to release for 24 h. Afterwards the PBS buffer was changed completely. The process was repeated twice. The solutions after 24, 48, and 72 h were analyzed photometrical with an infinite M200 reader from Tecan (Maennedorf, Switzerland). The absorbance of TiO_2_ at 300 nm was detected. Furthermore, the amount of TiO_2_ lost to the solution was observed with inductively coupled plasma atomic emission spectroscopy (ICP-OES) with a Ciros vision spectrometer from spectro (Kleve, Deutschland).

### Determination of the photoactivity

Photoactivity was measured by monitoring the decomposition of methylene blue as reported before (Fischer et al., 2014). Therefore, the gel was immersed in a 20 μM methylene blue PBS solution for 24 h in the dark to make sure the dye uptake of the gel was complete and equilibrium was established. A circle of a diameter of 1 cm was cut in the gels. Afterwards the gels were placed in 8 ml of a 20 μM solution of methylene blue and irradiated with a sunlamp (Heraus Original Hanau Suncare tanning tube 21/25 slim) for 2 h in total. For the first 40 min the concentrations were detected every 10 min, afterwards every 20 min. The concentrations were recorded with an infinite M200 reader from Tecan (Maennedorf, Switzerland).

### Thermal degradation

The thermal stability of the produced dried hydrogels was observed by thermos gravimetric analysis. A Pyris 1 TGA from Perkin Elmer was used to perform this measurement. Air was used as purge gas. The temperature range was 20–800°C with a heat rate of 10°C min^−1^.

### Cytotoxicity

Hydrogels were sterilized by autoclaving. Afterwards they were extracted in RPMI-1640 medium. A 1 ml solution was used per 3 cm2 hydrogel surface. The cell concentration was set to 6.7 × 10^4^ cells ml^−1^. Afterwards, 20 μl fetal bovine serum and 20 μl Alamar Blue were added to 180 μl of the cell suspension. The cells (L929, mouse fibroblasts) were bred over night at 37°C in a 5% CO_2_ atmosphere. After 24, 48, and 72 h the fluorescence of Alamar Blue at 590 nm was determined with an infinite M200 reader from Tecan (Maennedorf, Switzerland).

## Results and discussion

### Crystallinity of TiO_2_ nanoparticles

**Figure 2:**
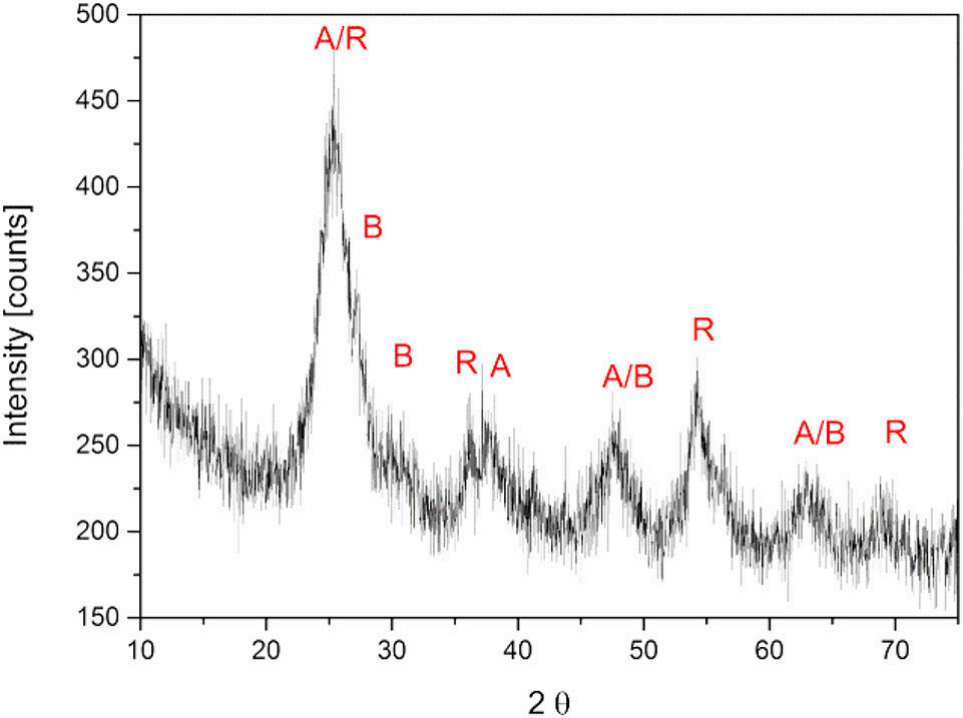
XRD pattern of crystallite TiO_2_ nanoparticles. The main reflexes were marked with A (anatase), B (brookite) and R (rutile).

The prepared TiO_2_ nanoparticle suspension was dried for 24 h at room temperature and crushed by mortar and pestle. The fine powder was analyzed by XRD to determine the crystal phases. The diffractogram is presented in Figure 2. The main reflexes were detected at 25.5°, 27.4°, 30.2°, 36.1°, 37.7°, 47.8°, 54.3°, 63.5°, and 69.2°. They were related to the main crystal phases of titania [anatase (Horn et al., 1972), rutile (Swope et al., 1995), and brookite (Meagher and Lager, 1979)] in Figure 2. Moreover, the weight fractions were calculated with equation (1) to result in a ratio of anatase 61%, rutile 19%, and brookite 20%. A high fraction of anatase like in this sample is reported to be favorable, because anatase is known to be the most photoactive crystal phase. (Zhang et al., 2014)

### Hydrogel appearance and optical properties

**Figure 3:**
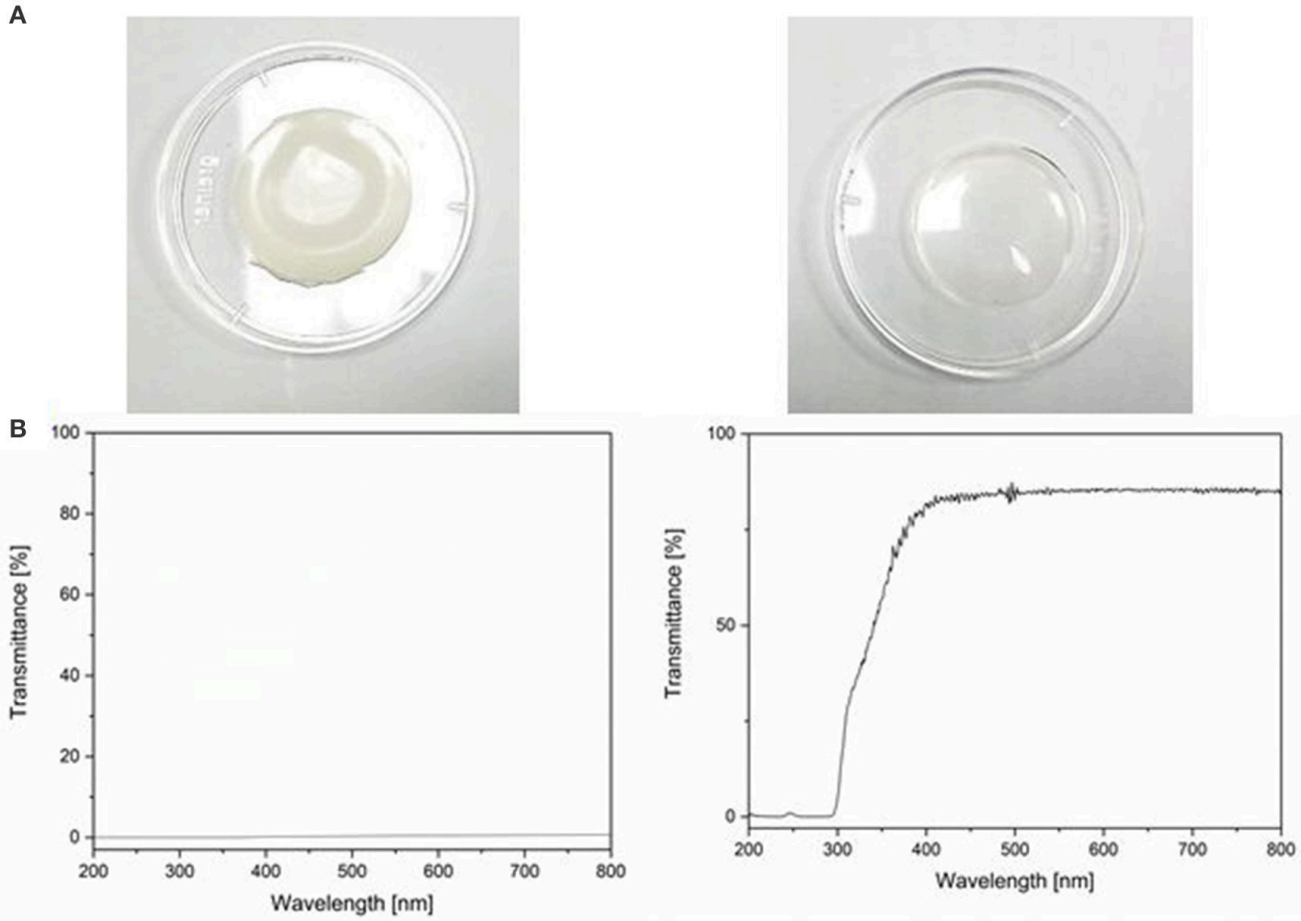
**(A)** Photo of hydrogels prepared with TiO_2_ nanoparticles as photoinitiator (left) and with the commercial photoinitiator α-HAP (right). **(B)** UV/VIS transmittance spectra of the aforementioned.

Figure 3A shows a picture of the dried hydrogels with TiO_2_ (left) and α-HAP (right) after photopolymerization. The TiO_2_ gels were about 1 mm thick and had a white, opaque, homogeneous, and shiny appearance. The diameter of the swollen gels was about 35 mm, while the dried gels had a diameter of 23 mm. The hydrogels prepared with the commercial photoinitiator α-HAP were transparent and had the same diameter and size like the hydrogels prepared with TiO_2_ as the photoinitiator. The white color of the TiO_2_-PEGDA-hydrogel was generated by the embedded TiO_2_. Therefore it can be assumed that TiO_2_ is homogeneously distributed in the gel.

The hydrogels synthesized with TiO_2_ as the photoinitiator were white and absorbed light in the entire range of visible light. In Figure 3B the UV/VIS transmittance spectra of both hydrogels are displayed. The transmittance of the hydrogel matrix is about 70% starting from 350 nm. The overall transmittance was lower than 1% when TiO_2_ was present in the hydrogel matrix.

### Mechanical properties

In Table 1 the rheological data at a shear frequency of 1 Hz are shown. Dynamic modulus (G^*^), loss modulus (G'), storage modulus (G”), and loss factor [tan(δ)] were determined. The complete graphs can be seen in Supplementary Figure 1. The moduli of the TiO_2_-PEGDA hydrogels were much smaller compared to the PEGDA hydrogels with the commercial initiator. This means on the one hand that the gels with TiO_2_ are much softer and less elastic (and therefore more viscous) than the gels with α-HAP. Higher softness could be beneficial for a medical application on tissue because the gels can adjust better with the tissue. On the other hand, an about three times smaller storage modulus (55.200 kPa compared to 131.333 kPa) corresponds to a larger mesh size (Lohmann et al., 2017) and a lower crosslinking density (Hajighasem and Kabiri, 2013). This influences the swelling ratio, too.

**Table 1:** Mechanical properties of the hydrogels. Rheological data were recorded at 1 Hz.

	**PEGDA hydrogel with TiO_2_**	**PEGDA hydrogel with α-HAP**
G*	55.2 kPa	131.3 kPa
G′	3.1 kPa	3.6 kPa
G″ tan(δ) q	55.2 kPa 0.057 392%	131.3 kPa 0.029 342%
tan(δ)	0.057	0.029
q	92%	342%

The swelling ratio of the TiO_2_-hybrid-hydrogels was 392 ± 28%. This is significantly higher than the swelling ratio of the gels made with the commercial photoinitiator (342 ± 3%.). This indicates higher mesh sizes and therefore confirms the results of the rheological characterization. A further possible reason for the higher swelling ratio is the increased hydrophilicity of the hydrogel due to the presence of superhydrophilic TiO_2_ nanoparticles (Wang et al., 1997, 1998; Gan et al., 2007). A higher swelling ratio is favorable for wound dressing applications because more wound exudate can be taken up and the dressing can be changed less often.

### Morphology

**Figure 4:**
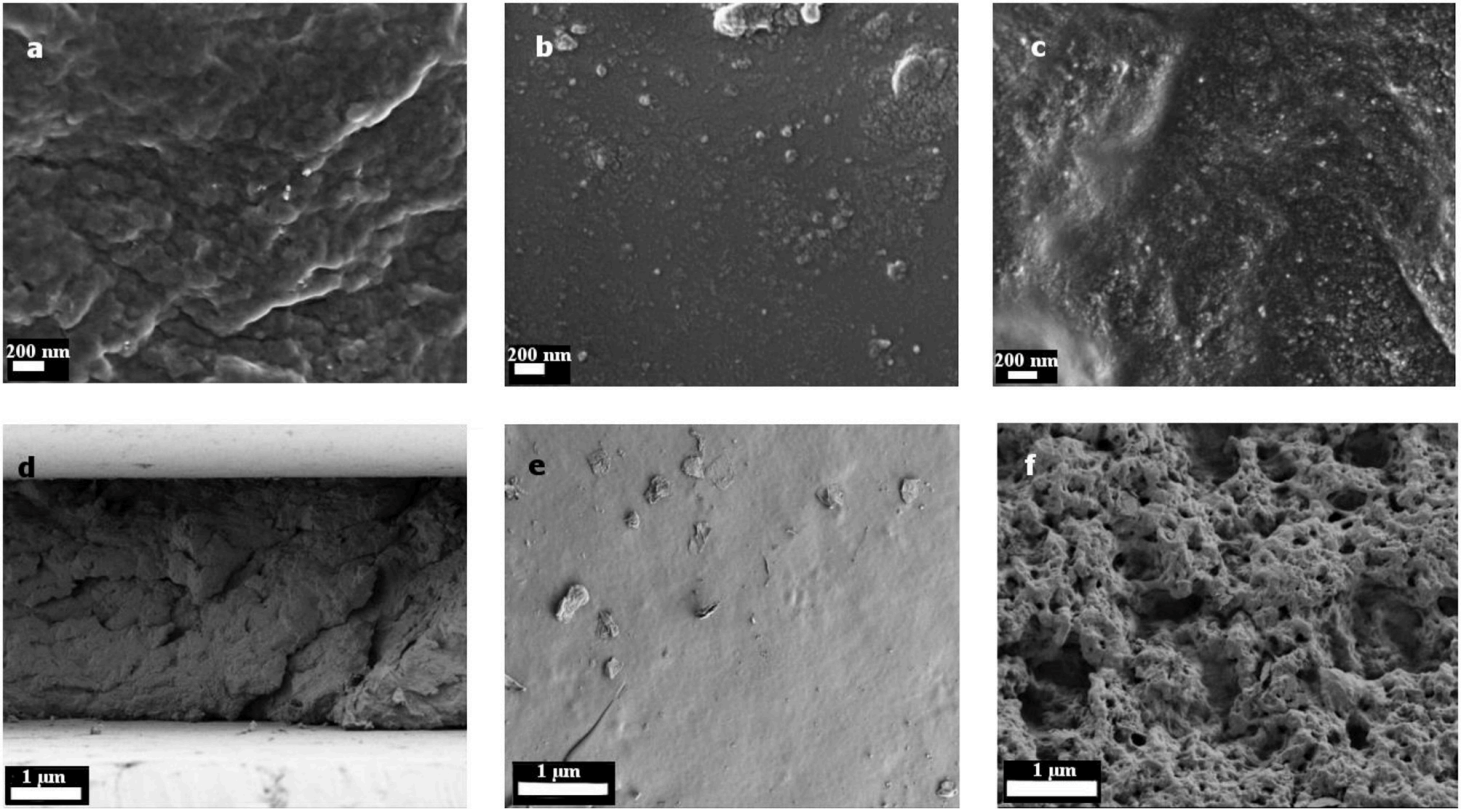
SEM images of the hydrogels with TiO_2_ as the photoinitiator. Pictures **(A)** and **(D)** show the cross section, **(B)** and **(E)** the top side and **(C)** and **(F)** the bottom side. In figures **(A)** to **(C)** the magnification is enlarged compared to figures **(D)** to **(F)**.

In Figure 4 the SEM images of the TiO_2_ gels are shown. The top side was the side which was facing the lamp during photopolymerization. This side of the gels (Figures 4B,E) was very smooth. The TiO_2_ nanoparticles were displaced as small, white spots in and on the surface. They had a size of about 10 to 20 nm. On the top side only some few particles can be seen (see Figure 4B). The main part of the TiO_2_ particles was finely distributed in the polymer matrix as it is demonstrated in Figure 4A and on the bottom side (Figure 4C). However, as can be seen in Figure 4C, there were some small aggregates found. The bottom side had a foam-like structure with several pores and holes. This can probably explain the higher swelling ratio compared to the smooth and dense gels with the commercial photoinitiator (Supplementary Figure 1), because those pores could encapsulate water. The hydrogels with α-HAP had a highly smooth top and bottom surface without any defects (Supplementary Figure 2).

### TiO_2_ release and photoactivity

In the release and photoactivity experiments only the hydrogels with TiO_2_ as the photoinitiator/photosensitizer were investigated because the hydrogels with the commercial initiator α-HAP contained no photoactive substance.

The loss of TiO_2_ nanoparticles is a main problem of many hybrid materials. If TiO_2_ is released from the material the photoactivity decreases. Furthermore, nanoparticles are under suspicion to cause Alzheimer's disease (Maher et al., 2016). Therefore, a loss of TiO_2_ in materials must be avoided for medical applications.

For this reason, a possible TiO_2_ release was investigated as described in section Release Study after 24, 48, and 120 h by UV/VIS spectroscopy and by ICP-OES, respectively. Neither in UV/VIS analysis nor in ICP-OES measurements was TiO_2_ detected. Consequently, the unwanted release of TiO_2_ nanoparticles can be excluded; the particles were embedded permanently within the polymer network of the hydrogel.

The photoactivity was monitored by degradation of methylene blue over 180 min. As discussed in section Morphology of top/bottom side of the gel differs. Therefore, the top and bottom sides of the hydrogels with TiO_2_ as the photoinitiator were investigated separately by turning each side up. The hydrogels were immersed in 20 μM methylene blue solution for 24 h in the dark.

**Figure 5:**
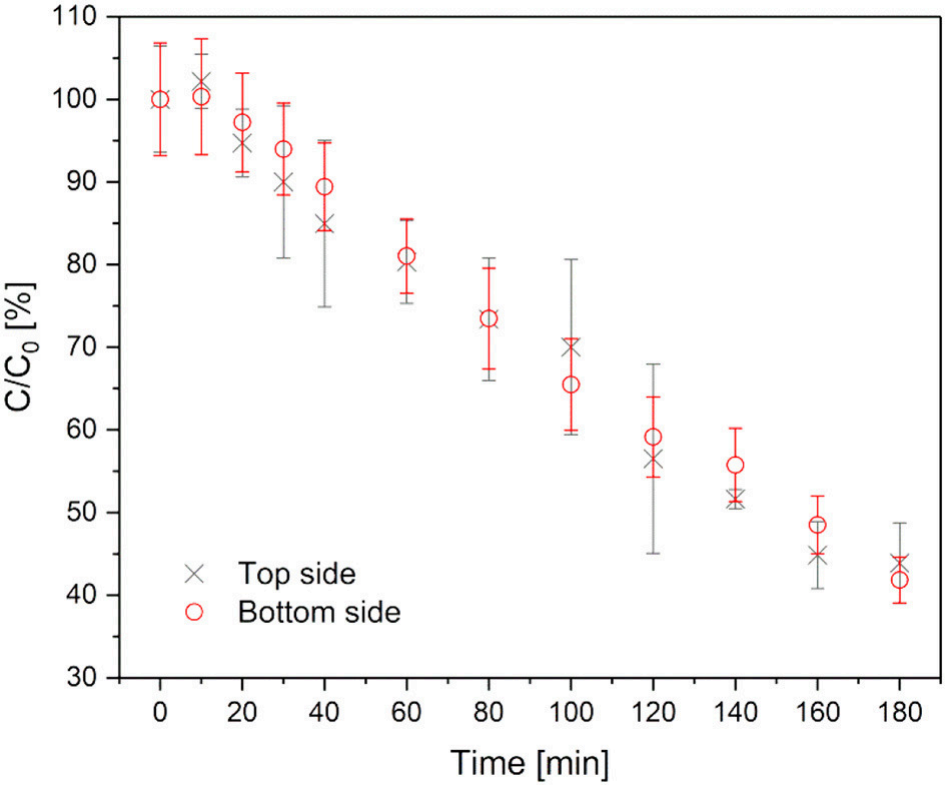
Methylene blue degradation in hydrogels with TiO_2_ as photoinitiator. Black crosses represent the degradation, when the top side is up, red circles when the bottom side is up.

The degradation of methylene blue is presented in Figure 5. In the first 10 min of irradiation a slight increase in the methylene blue concentration (about 5%) was detected. This can be explained by photodesorption as discussed for photochemical processes on TiO_2_ (Becerril et al., 2013). Afterwards, the photodecomposition takes place. Approximately 20% methylene blue was degraded per hour. The degradation rate stayed nearly constant over the whole time of observation. This indicates a zeroth order reaction, which is typical for reactions on catalytic surfaces. After 180 min about 60% of the methylene blue had been degraded. Furthermore, the degradation profile of the top/bottom sides was comparable. Consequently, the different surface morphology does not affect the photoactivity of the hydrogel. However, it should be considered that the morphology that was detected by SEM showed dried samples, while the hydrogels used in photocatalytic degradation tests were used in their swollen state. The recyclability of the hydrogels was not tested because they are meant for a one-time usage.

### Thermal stability

**Figure 6:**
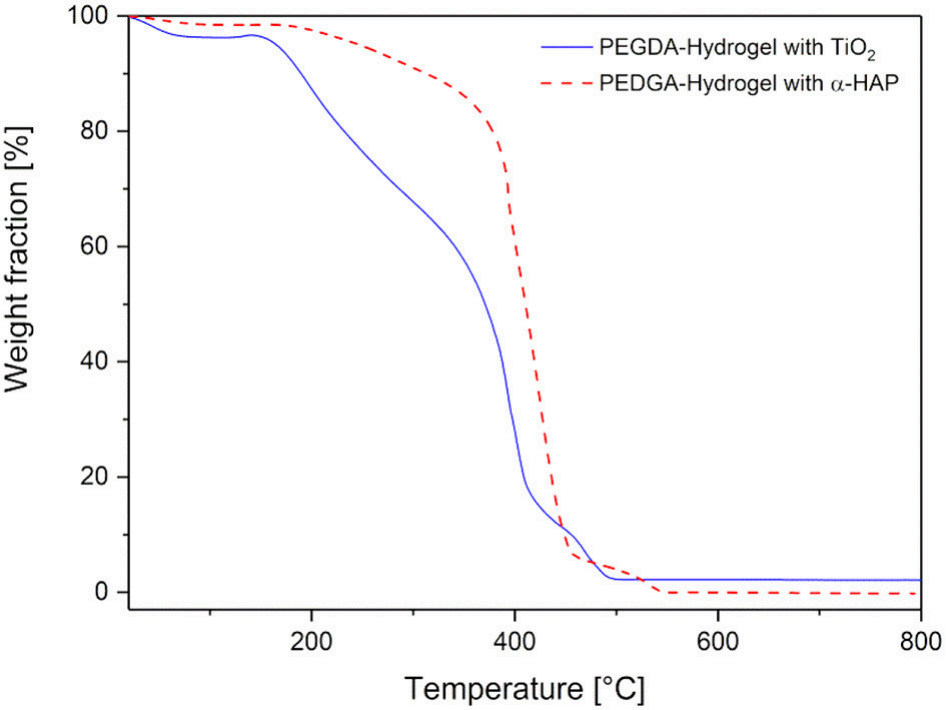
Thermogravimetric analysis of the hydrogels prepared with commercial photoinitiator α-HAP (red, dotted line) compared to the ones with TiO_2_ (blue, solid line).

The thermogravimetric analysis of the dried hydrogels is presented in Figure 6. Both hydrogel types showed a small weight loss of 2 and 4%, respectively, at a temperature of 100°C. This corresponds to a loss of water, which was absorbed under atmospheric conditions. Here, it can be concluded again, that the hydrogels with TiO_2_ have a slightly higher ability to absorb water (as already found for the swelling behavior in section Mechanical Properties). With increasing temperature, the weight of the hybrid gels stayed stable up to 150°C. This is very important to ensure a possible sterilization by autoclaving, which normally takes place at 121°C or 134°C. At higher temperatures thermal degradation was observed. First, there was a weight loss of about 35% up to a temperature of 330°C, followed by a degradation step of nearly 50% of the weight within the next 100°C. Above 450°C there was a forth weight loss of 5%. At 550°C the degradation was complete. There was a residual weight of about 2.2%, which corresponds to non-degradable TiO_2_. The degradation of the hydrogel that was produced with the commercial initiator was almost the same, except the second weight loss was smaller (just 15% up to 400°C). This means the hydrogels produced with the commercial photoinitiator were more stable at higher temperatures than the ones produced with TiO_2_. In all probability, the lower degradation temperature is induced by the lower crosslinking which was shown in section Mechanical Properties.

### Cytotoxicity

For biomedical applications like bandages or photodynamic therapy, cytocompatibility is very important. To prove this, the cytotoxicity was studied by investigation of the viability of mouse fibroblasts. The viability of the cells in the hydrogel extracts is shown in Table 2. It was not possible to investigate the viability directly on the gels, because cells cannot adhere to the very smooth surface. For that reason the cytotoxicity of the extracts of the hydrogels was monitored. Nearly 100% of the cells survived up to 72 h in the extracts. We found no indication of toxicity of the material from the above experiments such that we assume non-toxicity of the gel as well. Further studies will shed more light into this. This may be of relevance for future medical applications.

**Table 2:** Cell viability.

**Time [h]**	**PEGDA hydrogel with TiO_2_ (%)**	**PEGDA hydrogel with α-HAP (%)**
24	98	99
48	97	97
72	97	99

## Conclusion

In this study it was shown that nanosized crystalline TiO_2_ can be used as a photoinitiator for polymeric PEGDA hydrogels. The TiO_2_ used here consisted mainly of anatase. Using TiO_2_ as a photoinitiator is beneficial for medical applications because commercial organic photoinitiators (or their photocleavage products) are often toxic, while TiO_2_ is known to be biocompatible. The synthesized hydrogels have a high dynamic modulus (55.2 kPa at 1 Hz) and are therefore decidedly mechanically stable while still elastic (tan(δ) ≈ 0.06). Additionally, they can be sterilized by autoclaving due to their sufficient thermal stability. We found no indication of toxicity of the hydrogel material. These outstanding properties make the hydrogels very promising candidates for medical applications such as usage as a wound patch. Furthermore, the TiO_2_ was well distributed in the matrix and was still photoactive when irradiated with a sunlamp. Therefore, it can be used not only as a photoinitiator during polymerization of the hydrogels, but also as a photosensitizer in photodynamic therapy.

However, the photoactivity of the TiO_2_ in the hydrogels must be improved in further studies. To do so, the transparency of the hydrogels shall be increased. This will enable the activation of TiO_2_ below the polymer matrix and will allow the photosensitive material to be applied directly on a wound. Furthermore, additional biological tests like viability of further cell lines or antibacterial tests shall be done.

Nevertheless, TiO_2_ may have great potential as a photoinitiator in hydrogel materials for medical applications, especially because of the photosensitizing effect of the hydrogels with TiO_2_ which can be used in photodynamic therapy.

## Author contributions

BT performed and interpreted the experiments (rheology, TGA, hydrogel production and swelling ratio). SG planned, designed and performed particular experiments (UV/VIS and release studies), and wrote the manuscript. AS designed the experiment, gave scientific support, revised, and improved the manuscript. BA supervised the project and revised the manuscript.

### Conflict of interest statement

The authors declare that the research was conducted in the absence of any commercial or financial relationships that could be construed as a potential conflict of interest. The reviewer LT and handling Editor declared their shared affiliation.
