# Supplementary material for: TiO2 as Photosensitizer and Photoinitiator for Synthesis of Photoactive TiO2-PEGDA Hydrogel Without Organic Photoinitiator
Source: Front Chem. 2018 Aug 7;6:340. doi: 10.3389/fchem.2018.00340 (PMC6090817; doi:10.3389/fchem.2018.00340)
Supplement: Supplementary file 1 [file Data_Sheet_1.DOCX]

Supplementary Material

Production of Photoactive TiO_2_-PEGDA Hydrogel Without Organic Photoinitiator

Sarah Glass^1^, Betsy Trinklein^1^, Bernd Abel^1^, Agnes Schulze^1*^

^1^Leibniz Institute of Surface Engineering (IOM), Chemical Department, Permoserstrasse 15, D-04317 Leipzig, Germany

*** Correspondence:**Agnes Schulze
[agnes.schulze@iom-leipzig.de](mailto:agnes.schulze@iom-leipzig.de)


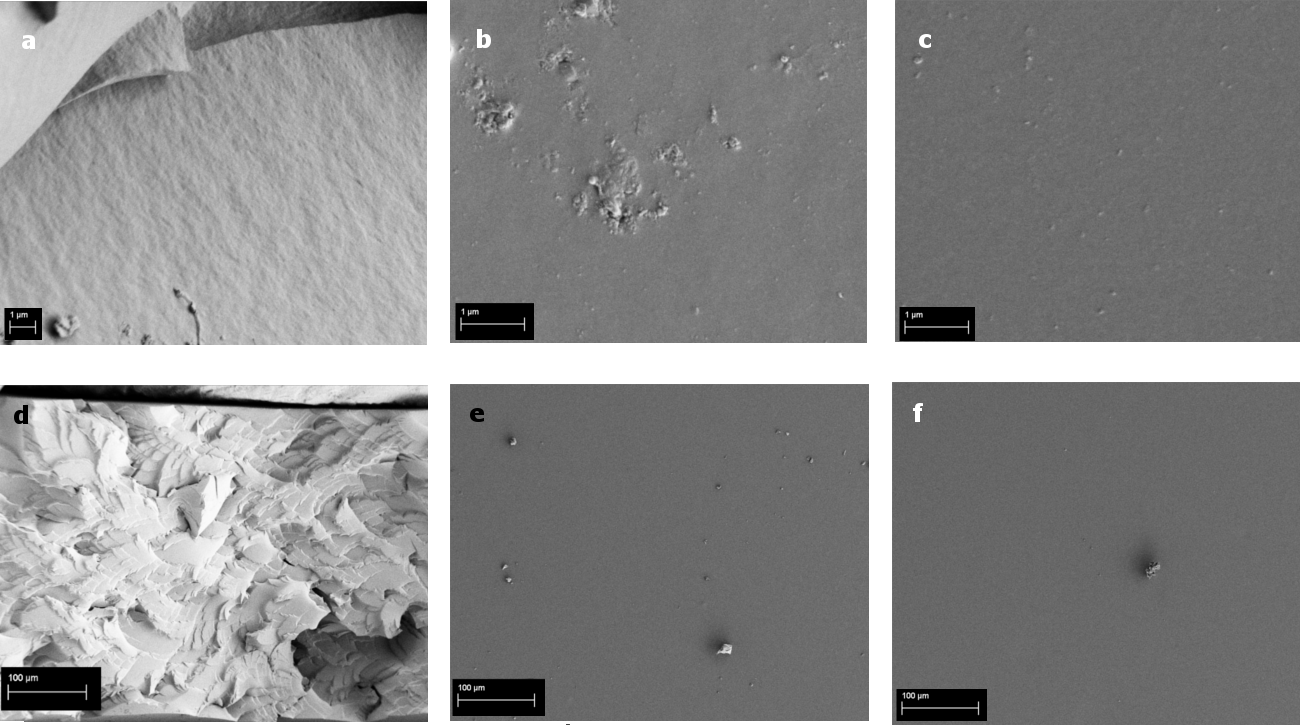


Supplementary Figure 2. REM images of the hydrogels with commercial initiator. Pictures a) and d) show the cross section, b) and e) the top side and c) and f) the bottom side. In figures a) to c) the magnification is enlarged compared to figures d) to f).
